# Targeting the Hippocampus in the Context of Trauma

**DOI:** 10.1016/j.bpsgos.2024.100335

**Published:** 2024-07-15

**Authors:** Sanne J.H. van Rooij

**Affiliations:** Department of Psychiatry and Behavioral Sciences, Emory University School of Medicine, Atlanta, Georgia

Transcranial magnetic stimulation (TMS) is a valuable tool for probing brain regions to investigate neural function (1). This approach can critically advance our understanding of the functions of the human brain and behavior by moving from observational neuroimaging studies to manipulating circuits and observing the effects on behavior. Originally, TMS was used to modulate the specific cortical region that was being targeted by either increasing or decreasing cortical excitability. Because of limitations of magnetic field penetration through the skull, subcortical areas deeper in the brain cannot be directly modulated by TMS. Leveraging increased understanding of cortical-subcortical brain networks, researchers have begun to use TMS to modulate subcortical areas through their functional or structural connections with cortical areas. To date, few researchers have attempted to use this method to understand behavioral mechanisms of psychopathology. The recent article by Webler *et al.* (2) is an important contribution to mechanistic probing of subcortical regions for neuropsychiatric conditions.

SEE CORRESPONDING ARTICLE NO. 100309 IN ISSUE 4/3

Researchers who have used TMS as a probe have predominantly studied the role of the prefrontal cortex (PFC) in neuropsychiatric disorders. This is consistent with clinical applications of TMS in psychiatry that have also centered around the PFC, predominantly the dorsolateral PFC, and Food and Drug Administration–cleared TMS treatments for neuropsychiatric conditions are targeting the PFC. Using TMS to probe other networks that could inform our understanding of neuropsychiatric symptoms through brain-behavior connections is critical to advancing the development of novel neurotherapeutics. Webler *et al.* (2) followed up on a small number of studies that probed the hippocampus to understand hippocampal-dependent learning and memory processes such as pivotal work from a decade ago (3).

The approach that Webler *et al.* (2) employed consists of the definition of a cortical target that is functionally connected with subcortical regions defined using resting-state functional connectivity. The left hippocampus was used as the seed, and the peak within the left posterior parietal cortex was defined with resting-state functional connectivity. Webler *et al.* (2) used precision functional mapping and electrical field modeling methods to define each individual’s stimulation target. Continuous theta burst stimulation (cTBS) (600 pulses in triplets) was delivered to the target following previous work that showed that hippocampal network–targeted TMS (HNT-TMS) using cTBS modulated hippocampal activation and augmented learning and memory processes (3).

The primary innovation of the work by Webler *et al.* (2) compared with earlier work is their goal to use HNT-TMS to modulate human fear learning and discrimination, a mechanism that has been consistently implicated in human anxiety and posttraumatic stress disorder (PTSD). To this end, college students with varying levels of PTSD symptoms were recruited for a 3-day study using a within-participant crossover design. On the first day, participants completed a clinical assessment and a magnetic resonance imaging scan to define the TMS target. On days 2 and 3, each of which occurred about 2 weeks after the other, participants received 2 cTBS sessions. One session included HNT-TMS and the other included vertex-TMS as a control. During these sessions, participants completed a fear-based and a neutral visual stimulus discrimination task, and both the TMS sessions and the order of the tasks were counterbalanced. It was expected that HNT-TMS would improve both fear and neutral discrimination.

The results of the study were not as straightforward as anticipated but nonetheless show promise for this approach. There were no main effects of HNT-TMS versus vertex-TMS on fear-based or neutral discrimination. Instead, HNT-TMS strengthened fear-based discrimination only in participants with lower fear sensitization. In other words, individuals who discriminated fear responses to novel stimuli better benefited more from HNT-TMS for fear-based discrimination. Webler *et al.* (2) were specifically interested in the hippocampal mechanism of pattern separation, which is the process of converting comparable events or experiences into distinct representations to promote discrimination, thereby facilitating contextualization of memories and behavior and preventing overgeneralization. Interestingly, the impact of HNT-TMS was only observed for fear-based discrimination and did not extend to the general pattern separation task that they applied. The study used a clever design and TMS approach to address an important question. The methods are rigorous, and the analyses are thorough. The results were clearly described, and while not in the expected direction and only significant for one condition, they will contribute to the currently sparse literature on hippocampal TMS.

There are several considerations and limitations of the research that deserve discussion, some of which were also raised by the authors. First, the sample size is modest (*n* = 25), and replication is needed. Second, the tasks used in the study include numerous layers and conditions, and it is possible that the single session of cTBS was not able to modulate the compound behavioral outcomes. Moreover, because the order of the tasks in the study was randomized, half of the participants performed the task after 1 cTBS session, and half completed it after 2 cTBS sessions. While the authors provide a rationale and cite papers showing that the effects of cTBS last for 50 minutes, there is much that is still unknown about the exact duration of the effects of stimulation, and this difference could have introduced variance and noise into the analyses. The use of functional neuroimaging after or combined with TMS to measure immediate effects of the stimulation on brain activation and circuit communication is an important future direction (1).

Another consideration is the use of cTBS and not intermittent TBS for strengthening hippocampal functioning. The authors provide a clear and robust rationale for using cTBS following previous work that showed that cTBS improved hippocampal-based learning and activation and functional connectivity (3); however, much is unknown about the exact effects of intermittent TBS or cTBS. The assumption that currently has the most support is that cTBS is comparable to slow repetitive TMS, thought to dampen cortical excitability. This is also presumed to depend on the dose, intertrial interval, and unknown individual differences. Future studies that investigate intermittent TBS and compare the effects with cTBS, hypothetically demonstrating opposing effects, are needed to better understand the potential of HNT-TMS.

Finally, it is important to note that most participants were female (*n* = 20, 80%), and the interval between sessions was about 2 weeks. Emerging research suggests the critical impact of menstrual cycle phase on fear conditioning and extinction, as well as PTSD symptoms, especially in the face of anxiety (4). This could have impacted the outcomes or introduced variability into this small sample that warrants further investigation. In our recent work, we showed sex differences in neural mechanisms of inhibition-related circuitry for the prediction of PTSD (5), further suggesting critical sex differences or sex-specific effects relevant to inhibitory processes. The findings here may not translate to the population at large, and sex differences or sex-specific effects and the consideration of menstrual cycle phase should be an important consideration for future work in this area.

Given the novelty of the work, the findings and considerations will provide important directions for future research. This study should be considered an important step toward the possibility of modulating the hippocampus to facilitate fear extinction processes with great clinical potential. The hippocampus is a key region for (episodic) memory and contextualization, which are critical to fear inhibition processes. There is particular interest in the hippocampus and fear extinction in the context of PTSD. Smaller hippocampal volume has consistently been identified as a neurobiological risk factor for PTSD symptoms and treatment nonresponse. Functionally, the hippocampus has been associated with PTSD, although neuroimaging studies have yielded findings in opposing directions. Lower hippocampal reactivity has been associated with PTSD in civilians with chronic and recent trauma exposure using threat processing, response inhibition, and contextual fear conditioning paradigms, whereas studies have shown greater hippocampal involvement during emotional memory encoding. Furthermore, in the study by Webler *et al.* (2), the hippocampus was targeted through the posterior parietal cortex. Interestingly, the left inferior parietal lobe has been implicated separately in PTSD trauma-focused therapy outcomes, such that lower left inferior parietal lobe engagement during contextual cue processing predicted poorer PTSD treatment outcomes (6). Impairments in behavioral measures of context processing and reduced activation in attentional control regions have been observed consistently in PTSD [e.g., (7)], and context processing has been suggested as a key deficit in PTSD (8). Contextualization of the trauma or emotional memory is a central component of trauma-focused therapy and has been postulated to be a key determinant in treatment success (9).

Future work that further investigates HPC-TMS to promote hippocampal functioning in context processing and fear discrimination is of great interest to the field. Clinical results may arise from the opportunity to combine HNT-TMS with exposure-based therapy to improve contextual processing and memory, thereby facilitating trauma-focused therapy (10). This is critical because 30% to 50% of patients with PTSD currently do not respond to exposure-based therapies, which causes great suffering and individual and societal burden. The finding that HNT-TMS was only beneficial for those with lower fear sensitization, while in need of further investigation and replication, has interesting implications for individualized treatment approaches, which is considered to be an important focus of the field and especially relevant to individualized targeting approaches. Determining the causal role of the hippocampus in specific functions has important implications for treatment. Building on the existence of functional networks, engaging deeper hippocampal targets in the brain is a promising strategy for probing subcortical regions and for treatment of neuropsychiatric disorders.
